# Alleviation of Reverse Salt Leakage across Nanofiber Supported Thin-Film Composite Forward Osmosis Membrane via Heat-Curing in Hot Water

**DOI:** 10.3390/membranes11040237

**Published:** 2021-03-27

**Authors:** Xiao-Xue Ke, Ting-Yu Wang, Xiao-Qiong Wu, Jiang-Ping Chen, Quan-Bao Zhao, Yu-Ming Zheng

**Affiliations:** 1 CAS Key Laboratory of Urban Pollutant Conversion, Institute of Urban Environment, Chinese Academy of Sciences, 1799 Jimei Road, Xiamen 361021, China; xxke@iue.ac.cn (X.-X.K.); tywang@iue.ac.cn (T.-Y.W.); xqwu@iue.ac.cn (X.-Q.W.); jpchen@iue.ac.cn (J.-P.C.); qbzhao@iue.ac.cn (Q.-B.Z.); 2 University of Chinese Academy of Sciences, 19A Yuquan Road, Beijing 100049, China; 3 CAS Center for Excellence in Regional Atmospheric Environment, Institute of Urban Environment, Chinese Academy of Sciences, 1799 Jimei Road, Xiamen 361021, China

**Keywords:** forward osmosis, thin-film composite, electrospun nanofiber, interfacial polymerization, hot-water-curing

## Abstract

Electrospun nanofiber with interconnected porous structure has been studied as a promising support layer of polyamide (PA) thin-film composite (TFC) forward osmosis (FO) membrane. However, its rough surface with irregular pores is prone to the formation of a defective PA active layer after interfacial polymerization, which shows high reverse salt leakage in FO desalination. Heat-curing is beneficial for crosslinking and stabilization of the PA layer. In this work, a nanofiber-supported PA TFC membrane was conceived to be cured on a hot water surface with preserved phase interface for potential “defect repair”, which could be realized by supplementary interfacial polymerization of residual monomers during heat-curing. The resultant hot-water-curing FO membrane with a more uniform superhydrophilic and highly crosslinked PA layer exhibited much lower reverse salt flux (FO: 0.3 gMH, PRO: 0.8 gMH) than that of oven-curing FO membrane (FO: 2.3 gMH, PRO: 2.2 gMH) and achieved ∼4 times higher separation efficiency. It showed superior stability owing to mitigated reverse salt leakage and osmotic pressure loss, with its water flux decline lower than a quarter that of the oven-curing membrane. This study could provide new insight into the fine-tuning of nanofiber-supported TFC FO membrane for high-quality desalination via a proper selection of heat-curing methods.

## 1. Introduction

Water reuse is a feasible and effective way to alleviate water scarcity caused by high population pressure and rapid economic growth. In recent years, membrane technology, a mild physical process, has become an important class of water treatment methods due to its high separation accuracy and satisfying effluent quality [1,2]. The forward osmosis (FO) process, inspired by the natural osmosis phenomenon, can spontaneously occur when driven by the osmotic pressure difference (i.e., concentration difference) across the selective semipermeable membrane. It emerges with its distinctive advantages of lower pollution tendency, higher resilience after cleaning and lower energy consumption than conventional pressure-driven membrane technologies. FO technology has shown intriguing potentials in various application fields, such as desalination, wastewater treatment, food and drug concentration, and power generation [3,4,5,6].

Currently, the primary challenge faced by FO technology is the qualified membranes for long-term practical applications [7,8]. The thin-film composite (TFC) membrane, which is composed of a dense polyamide (PA) active layer for selectivity and a porous support layer, is the mainstream of FO membranes. Typically, the ultrathin (hundreds of nanometers thick) PA active layer is synthesized by interfacial polymerization between two monomers in two immiscible phases, amine monomer (m-phenylenediamine, MPD) from the aqueous phase and acid chloride monomer (trimesoyl chloride, TMC) from the organic phase [9,10,11]. Conventionally, the support layer featured sponge- and finger-like pores is prepared via the phase-inversion method. The FO process can be operated under two modes, with the PA active layer facing feed solution (FO mode) or facing draw solution (PRO mode), respectively. The distinctive internal concentration polarization (ICP) during FO operation happens when the draw solution is diluted in the support layer by permeate water under FO mode or when the solutes from the feed solution are intercepted at the interface between the support layer and the inner surface of the active layer (PRO mode). Therefore, the phase-inversion support with tortuous and closed porous structure is prone to severe ICP due to the resistance to solute diffusion, and thus lead to a decrease in the concentration difference across the FO membrane and result in a decline of the effective osmotic driving force. The structural parameter (S = tτ/ε) was used to determine the mass transfer resistance of the support layer, which is related to its thickness (t), pore tortuosity (τ) and porosity (ε). In the past decade, electrospun nanofiber membrane with a lower S value, owing to its high porosity, open and porous structure, which can favor the mass transfer and the alleviation of ICP inside the membrane, has been emerged as an effective alternative to the traditional phase-inversion support [12,13,14,15].

Nonetheless, the structures and properties of PA layers are often affected by the beneath porous support during interfacial polymerization [16,17,18,19]. The phase-inversion support normally features a relatively smooth surface with small and uniform pore sizes, which is more favorable for the stable deposition of an active polyamide layer formed via interfacial polymerization. The wide pore size distribution and relatively rough morphology of the nanofiber support hinder the formation of stable defect-free PA layers to some extent, resulting in low salt rejection in desalination [20]. Current studies on nanofiber-supported PA-TFC FO membranes focus more on the amelioration of surface structure and property of the nanofiber support, such as electrospinning process control, nanofiber surface modification, nanomaterials incorporation, phase-inversion or coating interlayer construction [21,22,23,24,25,26,27]. Furthermore, separation performances of TFC membranes depend mainly on the structures and properties of PA active layers. As the PA layer is particularly important for the durable high-performance FO operation of an electrospun nanofiber-supported TFC FO membrane, more fundamental and deeper thoughts and studies on relevant influential factors need to be focused and implemented to improve the integrity and stability of PA active layers.

An interfacially polymerized PA active layer normally consists of crosslinked structures with amide bonds (−CO−NH−) produced by the main reaction of MPD and TMC and linear chains with carboxyl (−COOH) end groups derived from the side reaction between TMC and water. A higher crosslinking degree is demonstrated to be linked to a high rejection performance of TFC membranes [28,29,30]. Currently, the optimization on reaction conditions (monomer concentration, solvents, additives, time, temperature and humidity, etc.) during interfacial polymerization and post surface treatment of the as-made PA active layer, such as coating/grafting modification and solvent treatment, have been studied extensively for a better separation performance [31,32,33,34,35,36,37,38]. However, little research has focused on the effect of heat-curing on the PA layer formation, which was also reported to play an important role in fabricating less-defective PA layers with modified surface chemistry and structural properties. This could promote the production of crosslinked PA with continued polymerization within a suitable time at a moderately high-temperature. Meanwhile, residual solvents could evaporate quickly to terminate the interfacial reaction and avoid the formation of carboxylic PA.

Currently, there are two typical heat-curing methods applied to prepare PA active layers, hot-air-curing (oven-curing) and hot-water-curing. For example, Azizi et al. reported using an oven to heat-cured the PA layer at 80 °C for 10 min after interfacial polymerization, and the salt rejection of the resulting membrane possessing higher crosslinking was superior to that without heat treatment [39]. Zhan et al. Ghosh et al., and Han et al. respectively investigated the effects of curing conditions (temperature, time) on the morphologies and performances of the as-prepared PA-TFC membranes in nanofiltration (oven-curing), reverse osmosis (oven-curing) and FO (specific hot-water-curing) [40,41,42]. In addition, several studies demonstrated that conducting different heat-curing methods would make a significant difference in surface morphologies, physicochemical properties and separation performances of reverse osmosis membranes [43,44]. However, as almost all related studies were based on the phase-inversion support, none are on the electrospun nanofiber support. Though there may be no substantial difference in the PA layer formation with a specific heat-curing method using phase-inversion support or electrospinning support, essential studies on the effect of different heat-curing processes on the morphologies, properties and particularly the FO performances of nanofiber-supported TFC membranes are absent. Notably, Obaid et al. found that, compared to hot-air-curing and non-curing, hot-water-curing endowed the thin PA layer activated nanofibrous membrane with better hydrophilicity and higher oil/water separation performance. The authors also proposed that the residual MPD molecules absorbed in nanofibers bulk, or free in voids of nanofiber networks, could be able to continue to react sufficiently with TMC during heat-curing in the presence of water [45].

The TFC FO membrane needs an intact and dense polyamide selective layer. Moreover, research on hot-water-curing seldom notes that whether the membrane was immersed in hot water or in other ways. Inspired by previous literature, we conceived that floating the membrane on the surface of hot water might preserve the interface for possible further strengthened polymerization at high-temperature in the heat-curing process, and thus to promote the reaction between the residual active monomers to form smooth “patches” at the interface of the active and support layer, especially at possible defective sites of the polyamide layer. Specifically, two heat-curing methods (oven-curing and hot-water-curing) were employed to post-treat the PA layers constructed on the electrospun polyacrylonitrile (PAN) nanofiber support via interfacial polymerization. Surface morphology, roughness and hydrophilicity of the as-prepared TFC membranes were characterized by scanning electron microscope (SEM), atomic force microscopy (AFM) and water contact angle (WCA) analysis. X-ray photoelectron spectroscopy (XPS) and Fourier-transform infrared spectroscopy (FTIR) spectral measurements were applied to calculate the crosslinking degrees of the PA layers and their influences on the damage evolution behaviors after tensile tests were investigated. The structure–activity relationship between the as-prepared membranes with different heat-curing methods and their FO performances was explored with different draw solutes (NaCl or MgCl_2_). The stability of the nanofiber-supported TFC membranes was evaluated according to the flux decline after 16 h FO operation. Furthermore, possible formation mechanisms of the nanofiber-supported PA active layers post-treated by different heat-curing processes were proposed. This work aims to offer some references to the fine-tuning preparation of electrospun nanofiber-supported TFC FO membrane via a proper heat-curing process.

## 2. Materials and Methods

### 2.1. Materials and Chemicals

Polyacrylonitrile (PAN, M_w_ = 90,000 g/mol) was provided by Kunshan Hongyu Plastic Co. (Suzhou, China). Analytical grade N, N-dimethylformamide (DMF), N, N-dimethylacetamide (DMAC), sodium chloride (NaCl), magnesium chloride (MgCl_2_·6H_2_O) and hexane were obtained from Sinopharm Chemical Reagent Co. Ltd. (Shanghai, China). Chemicals used in PA formation: MPD (>99%) and TMC (98%) were purchased from Sigma-Aldrich (St. Louis, MO, USA). All water used in this work was deionized (DI) water.

### 2.2. Membrane Preparation

**Figure 1:**
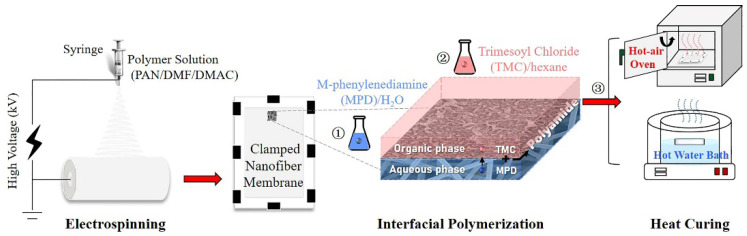
Schematic diagram of the preparation process of nanofiber-supported thin-film composite (TFC) forward osmosis (FO) membranes via different heat-curing methods.

The preparation process of the nanofiber-supported TFC FO membrane is shown in Figure 1, which was divided into three steps: (1) electrospinning of PAN nanofiber support layer according to our previous work [12]; (2) interfacial polymerization of the PA active layer; (3) heat-curing (oven-curing/hot-water-curing).

In detail, an ultrathin PA active layer was synthesized on the PAN nanofiber support via interfacial polymerization between MPD and TMC. The aqueous MPD and organic TMC solutions were freshly prepared in brown screw-capped bottles just before use by stirring and ultrasonication at room temperature in the dark. First, the nanofiber support membrane (11 cm × 15 cm) was clamped between a Teflon plate and a frame. 2% (*w/v*) MPD solution was poured on the top surface of the membrane to allow submersion for 2 min. Afterward, excess MPD solution was drained from the support surface, and the wet support was taken out to exclude air bubbles within the membrane and achieve a proper surface state using an air knife. Then, the nanofiber support was fixed again, and 0.15% (*w/v*) TMC in hexane was poured onto the upper surface of the support for about 1 min to allow complete reaction. Finally, the obtained TFC membrane was prepared (1) without curing; (2) curing in a hot-air oven at 80 °C for 10 min; and (3) curing by floating on a hot water bath (60, 70, 80, 90 °C) for a certain time (2, 4, 6, 8, 10 min). All the membranes were stored in DI water at 4 °C prior to use, and the non-curing, oven-curing and hot-water-curing membranes were defined as n-TFC, o-TFC and w-TFC, respectively.

### 2.3. Membrane Characterization

The surface and cross-section morphology of all the as-prepared nanofiber-supported TFC membranes via different heat-curing processes were observed with a field emission scanning electron microscope (FESEM, S-4800, Hitachi, Tokyo, Japan) at an accelerating voltage of 5 kV. All samples were sputter-coated with a thin gold layer for better imaging. The ethanol wetted membranes were frozen in liquid nitrogen for a fast brittle fracture to obtain a smooth cross-section. Images of epoxy resin-embedded and sectioned ultrathin slices of the cross-sectional structure of the prepared TFC membranes were captured by transmission electron microscope (TEM, Talos-S, FEI, Hillsboro, OR, USA) under an operating voltage of 100 kV. The fine microstructure and surface roughness (average roughness: R_a_; root-mean-square roughness: R_q_; maximum roughness: R_max_) of the PA layers were characterized by AFM (Dimension Icon, Bruker, billerica, MA, USA) in noncontact mode over the projection area of 5 µm × 5 µm. Dynamic WCA on the surface of the active layers and nanofiber supports over time (s) were determined by a sessile drop method using a contact angle tester (Precise Test, Dongguan, China) at room temperature, and the volume of a water droplet was fixed at 2 μL. The surface atomic compositions of PA layers were analyzed by applying XPS (Axis Supra, Kratos, Manchester, Britain). The chemical functional groups of all membranes were scanned under Transmission mode using FTIR (iS10, Thermo, Waltham, MA, USA) in the wavenumber range of 400–4000 cm^−1^ with a resolution of 2 cm^−1^, and the number of scans was 16. The tensile tests of TFC membranes were conducted on a universal tensile machine (AGS-X, Shimadzu, Kyoto, Japan). The thicknesses of the membranes were measured using an electronic thickness gauge, and a span length of 10 cm (L_0_ = 10 cm) and a crosshead speed of 10 mm/min were employed for all the tests.

### 2.4. Forward Osmosis Performance

A lab-scale FO system (as shown in Figure S1) containing a self-made membrane module with an effective membrane area of 33.56 cm^2^ was employed to evaluate the performances of the as-fabricated FO membranes prepared with different heat-curing methods. The temperatures of feed and draw solutions were kept at 25 °C. Simulated seawater 3.5 wt % NaCl and 0.5 M MgCl_2_ were used as draw solutions. The initial volumes of feed solution (DI water) and draw solutions were fixed at 1 L and were co-currently circulated with a fixed crossflow velocity of 8.3 cm/s using peristaltic pumps (WT600, Longer, Baoding, China). The feed solution was successively magnetic stirred to keep its homogeneity. The weight increment of the draw solution and the salt concentration in the feed solution was monitored by a digital weight balance (SF6001F, Ohaus, Parsippany, NJ, USA) and a conductivity meter (CON110, Eutech Instruments, Singapore), respectively, and were both connected to a computer with data logging systems. FO tests were conducted under both FO mode (active layer facing the feed solution) and PRO mode (active layer facing the draw solution). Each membrane sample was stabilized for 0.5 h before data recording.

Water flux (*J_w_*, L m^−2^ h^−1^, abbreviated as LMH) and reverse salt flux (*J_s_*, g m^−2^ h^−1^, abbreviated as gMH) of the as-fabricated TFC membranes were calculated by the following Equations (1) and (2), respectively:

(1) $$J_{w}=\frac{\Delta V}{A\Delta t}$$

Where Δ*V* (L) is the volume of permeate water collected in the draw solution after Δ*t* (h), and *A* is the effective membrane area (m^2^):

(2) $$J_{s}=\frac{C_{t}V_{t}-C_{i}V_{i}}{A\Delta t}$$

where *C_t_* (mg/L) and *V_t_* (L) are salt concentration and volume of the feed at time *t* (h), respectively; *C_i_* (mg/L) and *V_i_* (L) are the initial salt concentration and volume of the feed solution, respectively.

### 2.5. Determination of Intrinsic Transport Parameters

Water permeability coefficient (*A*), salt permeability coefficient (*B*) of o-TFC and w-TFC membranes were calculated with “Excel Spreadsheet for Determination of FO Membrane Parameters” shared by Tiraferri et al. [46]. Specifically, pure water flux and reverse salt flux in each stage were acquired from a four-stage FO experiment with a gradient concentration of draw solutions (0.5, 1, 1.5, 2 M NaCl).

## 3. Results and Discussion

### 3.1. Surface Morphology

Generally, in the interfacial polymerization process, aqueous MPD monomers absorbed in the porous support can diffuse across the two-phase interface and react with TMC in the organic solution above the support surface. The formed rough PA film features a large number of balloon-like nodules due to the gas nanobubbles trapped between the PA layer and support layer escaped [30]. Moreover, then the diffusion of MPD monomers into the organic phase could be hindered by the as-formed PA film, which is called the “self-inhibiting effect”, which would bring about smoother PA structures. Finally, swollen balloon-like nodules would partly deflate into leaf-like structures with solvent evaporation.

**Figure 2:**
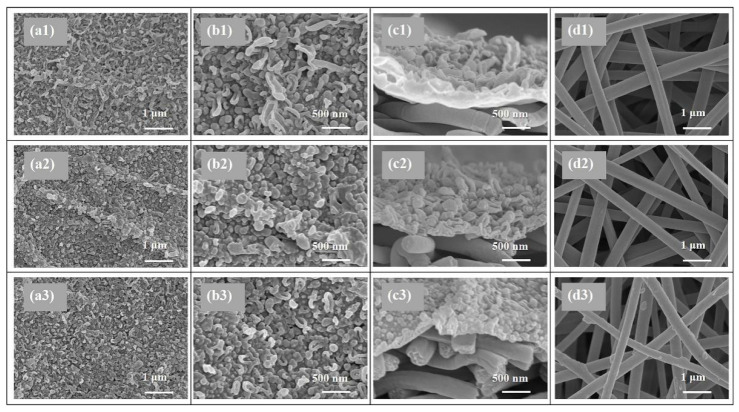
SEM images of the nanofiber-supported TFC FO membranes prepared via different heat-curing methods: (**a1–d1**) n-TFC; (**a2**–**d2**) o-TFC; (**a3**–**d3**) n-TFC; (**a1–3**,**b1–3**) active layer; (**c1–3**) cross-section; (**d1–3**) support layer.

SEM and AFM analysis were conducted to investigate the surface morphology of the TFC FO membranes prepared via different heat-curing methods. According to the SEM observations (Figure 2a,b), all the active layers synthesized via interfacial polymerization featured typical ridge-valley surfaces with randomly stacked nodular or leaf-like structures. As shown in Figure 2a1,b1, the PA layer dried in ambient air without heat-curing presented disordered surface morphology dominated with irregular wood-ear-like structures attributed to the quite slow solvent evaporation. More balloon-like nodules were observed on the active layers of o-TFC (Figure 2b2) and w-TFC (Figure 2b3) membranes with further enhanced polymerization and timely solvent volatilization in the heat-curing step.

Moreover, oven-curing was prone to generate an undulating PA layer (as shown in Figure 2a2) with quite fast solvent evaporation in hot air, as the aqueous/organic interface collapsed quickly, the as-formed PA chains could cave into the substrate pores, and the residual reactive monomers could mix randomly through the defective sites of the PA film. In comparison, when membrane heat-cured on hot water, the PA layer of w-TFC exhibited finer and more uniform nodular structures (Figure 2a3) for prolonged and enhanced interfacial polymerization with retained aqueous/organic interface. It is worth noting that there were some thin PA coatings and filaments on the nanofibers after hot-water-curing (Figure 2d3), which could be beneficial to strengthen the nanofiber support, and the interlayer binding between the support and the PA active layer.

**Figure 3:**
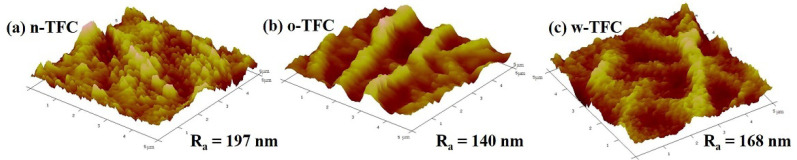
Atomic force microscopy (AFM) images of the nanofiber-supported TFC FO membranes prepared via different heat-curing methods.

The values of surface roughness acquired from AFM analysis could further quantify the microstructure features of the PA layers observed in the SEM images. Specific roughness values of R_a_, RMS, and R_max_ are summarized in Table 1. The approximate average roughnesses (R_a_) of the non-curing, oven-curing and hot-water-curing nanofiber-supported PA layers are 197, 140 and 168 nm, respectively. However, as shown in Figure 3, the PA active layer of o-TFC concaved into the voids among nanofibers and showed the most apparent nanofiber skeleton, as the reaction interface disappeared and the solvent evaporated rapidly during oven-curing. In contrast, a more uniform hot-water-curing PA layer of w-TFC covered the nanofiber support due to the retained two-phase interface when the membrane floated on hot water. The AFM results corresponded with observations of the magnified cross-section images of the PA layers (Figure 2c).

**Figure 4:**
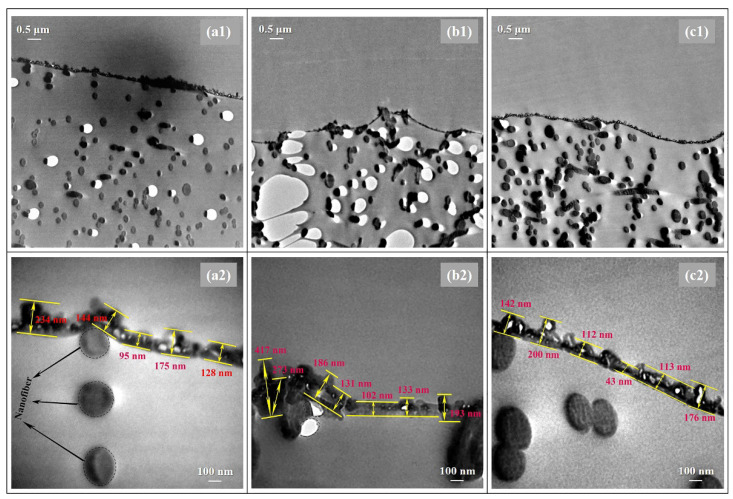
TEM images of the cross-section morphologies of the nanofiber-supported TFC FO membranes prepared via different heat-curing methods: (**a1**,**a2**) n-TFC; (**b1**,**b2**) o-TFC; (**c1**,**c2**) w-TFC.

In addition, TEM analysis was employed to deep study the thicknesses and morphologies of the PA layers prepared via different heat-curing methods. As shown in Figure 4, distinctly, the non-curing n-TFC showed an irregular PA surface with thicknesses of 95–234 nm, and the PA layer microstructures of oven-curing o-TFC tended to be concaved into the voids of the nanofiber support and featured a relatively wide thicknesses distribution from 102 to 417 nm, while the w-TFC PA layer exhibited the finest microstructures within 100–200 nm thicknesses range. Overall, the TEM results were in good accordance with SEM and AFM analysis.

### 3.2. Chemical Analysis

In addition to the effect on the surface morphologies, heat-curing processes could also significantly affect the chemical properties of the nanofiber-supported PA layers. The difference between the surface and bulk chemical structures of the polyamide layers can be evident by the combined XPS and FTIR analysis, respectively.

Complete interfacial polymerization of TMC (acyl chloride, −COCl) and MPD (amine group, −NH_2_) produced crosslinked PA with amide bonds (−CO−NH−), while insufficient polymerization generated linear PA with carboxylic end group (−COOH) originated from hydrolysis of a residual acyl chloride with water.

**Figure 5:**
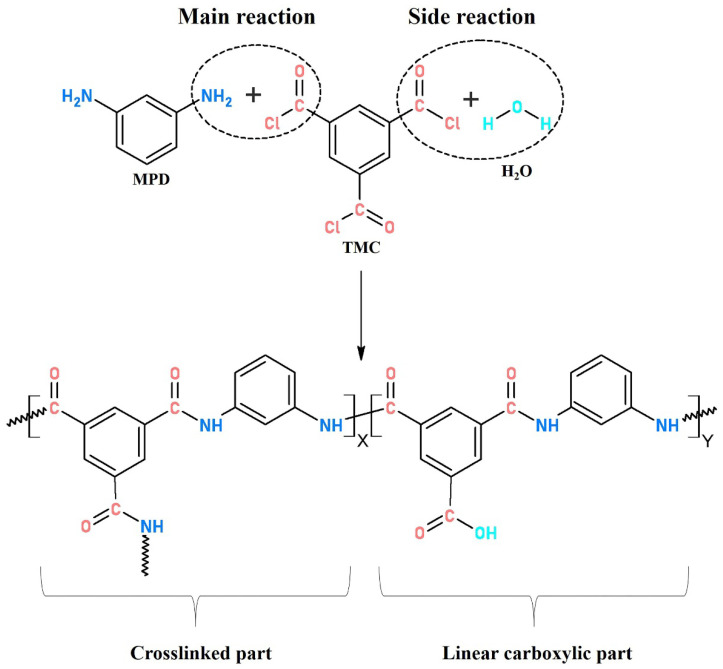
Typical structure of interfacially polymerized polyamide.

Crosslinking degree of the PA layer can be calculated according to Equations (3) and (4):

(3) X+Y=1

(4) $$\;\;O/Nratio=\frac{3X+4Y}{3X+2Y}$$

where *X* and *Y* represent the proportion of crosslinked and linear structure in interfacially polymerized PA, respectively (as shown in Figure 5); O/N ratio in PA layer can be evaluated from atomic compositions of XPS results.

**Figure 6:**
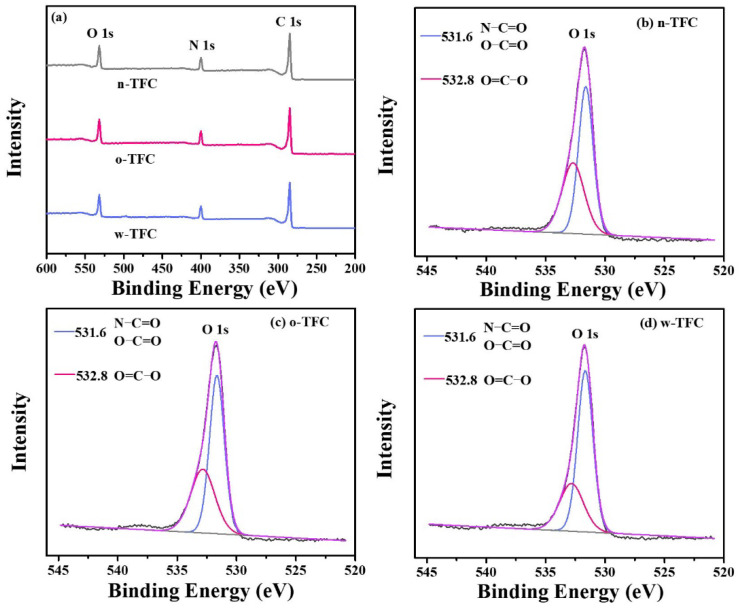
(**a**) The ray photoelectron spectroscopy (XPS) wide-scan spectra of TFC membranes prepared via different heat curing methods; high-resolution O 1s spectra of (**b**) n-TFC; (**c**) o-TFC; (**d**) w-TFC FO membranes.

The atomic compositions determined according to the intensity values of C 1s (284.8 eV), O 1s (531.8 eV), and N 1s (399.8 eV) peaks in XPS wide-scan spectra (Figure 6a), calculated O/N ratio and crosslinking degree (*X*/*Y*%) of the PA films synthesized via different heat-curing methods, are summarized in Table 2. As shown in Table 2, compared with the non-curing n-TFC membrane (O/N ratio: 1.24; *X*/*Y*%: 68.11%), the lower O/N atomic ratio and higher crosslinking degree of o-TFC (O/N ratio: 1.22; *X*/*Y*%: 70.55%) and w-TFC (O/N ratio: 1.13; *X*/*Y*%: 81.27%) membranes indicated that further enhanced reaction in the heat-curing procedure produced PA layers with higher amide contents. Obviously, hot-water-curing was beneficial for forming a highly crosslinked PA layer on the nanofiber support, while oven-curing brought about looser active layers, which was attributed to the complete polymerization when the membrane floated on a hot water bath with a maintained two-phase interface in the heat-curing procedure.

To further reveal the effect of the heat-curing process on the chemical state of the surface elements in the nanofiber-supported PA layer, the binding energy shifts of deconvoluted O 1s peak were investigated. The high-resolution O 1s spectra can be divided into two peaks: (i) carbonyl oxygen (O=C‒O/O=C‒N) with the binding energy at 531.6 eV and (ii) carboxylic oxygen (O=C‒O) with the binding energy at 532.8 eV [45]. A lower carboxylic/carbonyl ratio implies that crosslinking structures dominate in the PA layer.

High-resolution O 1s spectra of TFC membranes prepared via different heat-curing methods are shown in Figure 6b–d, and curve-fitted results of two peak components are summarized in Table 3. Notably, the PA active layer dried in ambient air without heat-curing tended to get much more carboxylic structures with a carboxylic/carbonyl ratio of 0.74, since residual water in support evaporated much slower than hexane on PA surface, and unreacted TMC were more likely to react with water. Despite the enhanced reaction of residual monomers in the oven-curing process, simultaneously accelerated evaporation of hexane in residual organic phase on the PA surface and relatively slower removal of water in bulk or voids of nanofiber support could also facilitate the hydrolysis of TMC monomer due to the gradual collapse of micro-interfaces with solvents evaporation. Thus, the higher proportion of carboxylic acid component and less crosslinked portion with a ratio of 0.66 in the formed PA layer of o-TFC can be verified. Additionally, when the nanofiber-supported PA layer was heat-treated above a hot water bath, continued crosslinking of PA chains on the nanofiber support could be promoted by maintaining a two-phase interface to obtain an active layer with fewer defects, which could be indicated by the lower calculated carboxylic/carbonyl ratio of 0.47.

**Figure 7:**
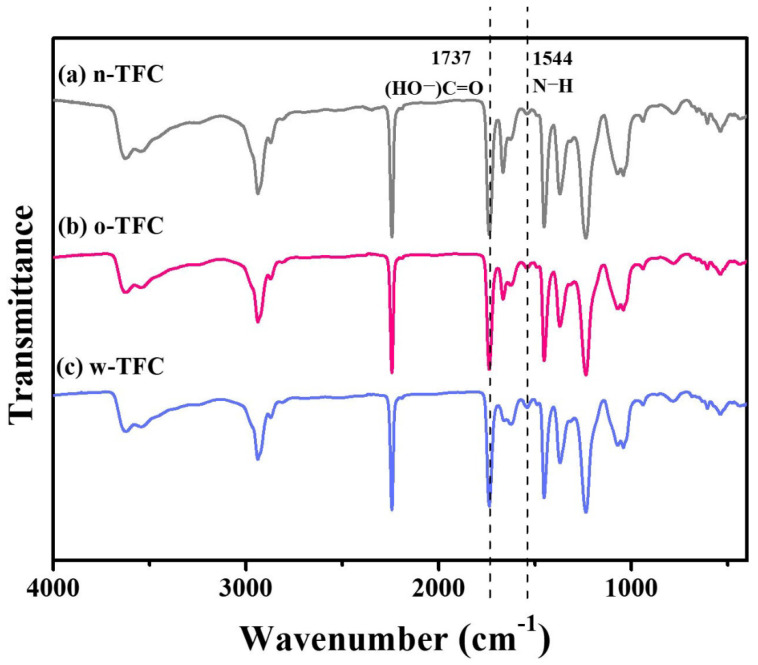
FTIR spectra of the nanofiber-supported TFC FO membranes prepared with different heat-curing methods.

FTIR spectra of the nanofiber-supported TFC FO membranes prepared via different heat-curing methods are shown in Figure 7. The characteristic spectra of all the membranes clearly confirmed the successful synthesis of PA: vibration peak of −COCl at 1763 cm^−^^1^ disappeared; the characteristic peak of −CO−NH− at 1662 cm^−1^ and 1544 cm^−^^1^ attributed, respectively, to stretching vibration of C=O amide I and N-H amide II the crosslinked PA appeared; peak at 1737 cm^−1^ was relevant to stretching vibration of C=O belonged to the linear carboxylic PA. Furthermore, slight differences among peak intensities of n-TFC, o-TFC and w-TFC membranes attributed to the PA layers indicated the effects of different heat-curing methods on the interfacial polymerization processes on the nanofiber supports, which could be identified by the comparison of I_1544_/I_1737_ ratios according to peak intensity at 1544 cm^−1^ (N–H) and 1737 cm^−1^ (HO−C=O) of FTIR spectra [29,43]. The specific values are summarized in Table 4. The highest **I_1544_/I_1__737_** ratio of 0.141 demonstrated that the most complete interfacial polymerization between TMC and MPD after hot-water-curing contributed to more crosslinked PA with more amide contents, which was consistent with the XPS results mentioned above.

As a result, clear differences of O/N atomic and carboxylic/carbonyl oxygen ratios of n-TFC, o-TFC and h-TFC revealed from XPS analysis along with I_1544_/I_1737_ ratio from FTIR spectra suggested that conventional oven-curing might be prone to get a looser and defective active layer on nanofiber substrates with less dense crosslinking PA networks. Moreover, the results showed that heat-curing above hot water could be considered as a chemical modification process with the ability to enhance the crosslinking degree of the PA layer supported by the nanofiber membrane and thus contribute to a higher rejection to salt ions in FO application.

### 3.3. Surface Hydrophilicity

**Figure 8:**
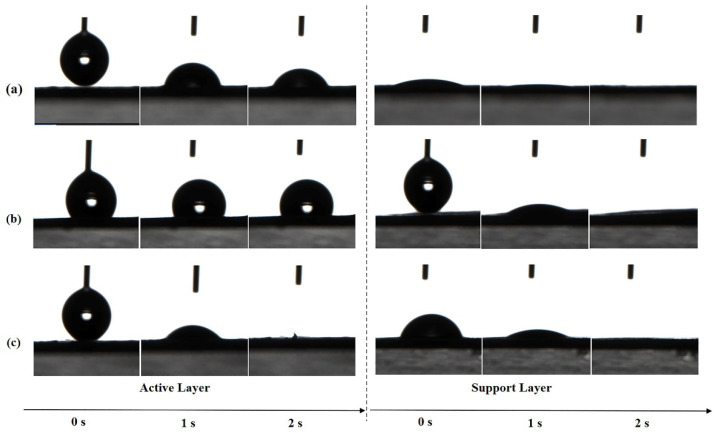
The changes of water contact angles (WCA) of the nanofiber TFC FO membranes prepared with different heat-curing methods over time: (**a**) n-TFC; (**b**) o-TFC; (**c**) w-TFC.

The absorption of water molecules on the membrane surface is driven by hydrophilic groups in the chemical structure of PA and is affected by the surface morphology and roughness. The surface hydrophilicity of both layers of TFC FO membranes prepared by different heat-curing processes was evaluated by determining the contact angles for deionized (DI) water. As shown in Figure 8, the active layer of w-TFC with more uniform surface microstructures was completely wetted by the water drop within 2 s, which indicated superhydrophilicity, while the water contact angle of o-TFC stayed above 90° after 2 s, which was related to its surface morphology and roughness observed in SEM and AFM analysis. Notably, all the supports showed excellent superhydrophilicity at 2 s, which mainly depended on the property of the laminated electrospun PAN nanofiber essentially, though there are slight differences in the wetting processes attributed to the unavoidable inhomogeneity of nanofiber membrane from the electrospinning process.

### 3.4. Mechanical Property of the Polyamide Layer

**Figure 9:**
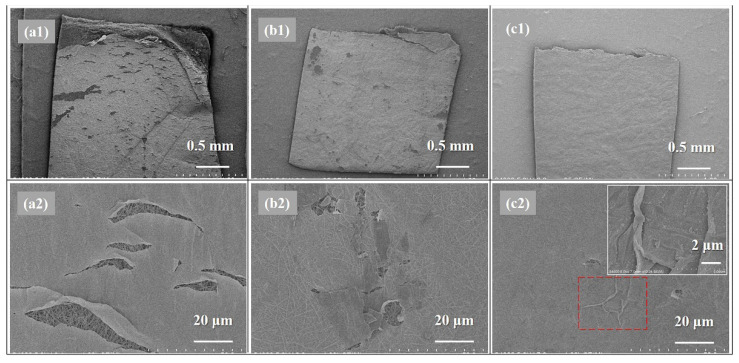
SEM images of the damaged nanofiber TFC FO membranes: (**a1**,**a2**) n-TFC; (**b1**,**b2**) o-TFC; (**c1**,**c2**) w-TFC (inserted image: magnification of “wrinkle” in rectangle with a red dotted line).

As the mechanical property of a TFC membrane was almost determined by the strength of the support layer, SEM morphologies of the PA active layers of as-prepared TFC FO membranes after tensile tests were investigated for further study on the effects of different heat-curing processes on the mechanical properties of the obtained nanofiber-supported PA layers, which are relevant to the crosslinking degree according to above discussion of XPS and FTIR results. As can be seen in Figure 9, it can be concluded that w-TFC with the highest crosslinking degree exhibited the best mechanical strength and ductility in the tensile test. The SEM images of damaged w-TFC, the PA layer with fewer cracks wrinkled (as shown in the insert image of Figure 9c2) for resistance to the increasing tensile force, while there were more significant cracks (Figure 9b2) on the active layer of o-TFC, and the cracks extended along the stretching direction. Obviously, many large cracks on the non-curing PA layer showed that the n-TFC had the lowest strain and was prone to seriously break (Figure 9a2) after stretching. Above all, the high crosslinking degree of the nanofiber-supported PA layer benefited from the further and complete interfacial polymerization in the heat-curing process, especially when floating on the hot water surface, which could be an effective way to enhance the mechanical property of the PA layer. Thereby, the hot-water-curing PA-TFC membranes based on nanofiber supports would be expected to serve as stable and durable FO membranes with stronger active layers.

### 3.5. FO Performance

As reported, a highly crosslinked PA network possesses a smaller free volume [27], which could relieve the reverse salt diffusion and mitigate the decline of effective osmotic pressure. Pure water flux (*J_w_*) and reverse salt flux (*J_s_*) were evaluated under FO, and PRO modes withdraw solution of simulated seawater (3.5 wt % NaCl) and thus investigating the effect of different heat-curing methods on the FO performances of the as-prepared nanofiber-supported TFC membranes with PA active layers of different crosslinking degrees. The optimal hot-water-curing conditions (temperature and time) were determined as 80 °C and 5 min according to the results shown in Figure S2a,b.

**Figure 10:**
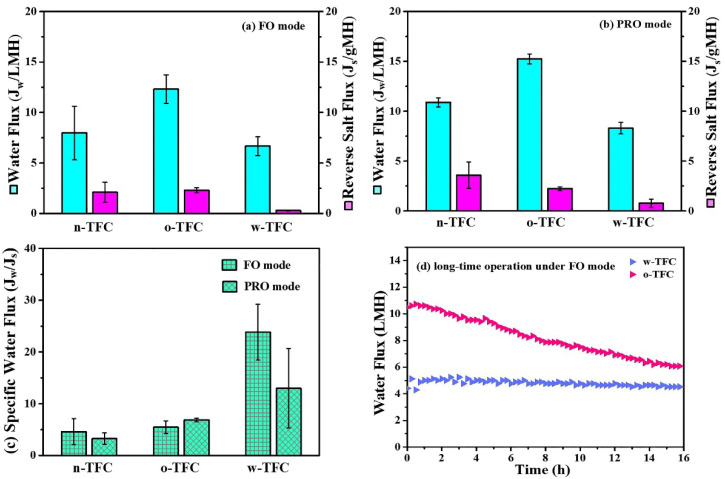
Water flux (*J_w_*) and reverse salt flux (*J_s_*) of the nanofiber-supported TFC FO membranes prepared with different heat-curing methods: (**a**) FO mode; (**b**) PRO mode; (**c**) calculated specific water flux (*J_w_*/*J_s_*, L/g) under two modes. (**d**) Comparison of water flux changes of the nanofiber TFC FO membranes via hot-water-curing and oven-curing over time under FO mode (Feed solution: DI water; Draw solution: 3.5 wt % NaCl).

Figure 10 displayed the FO performances of the nanofiber-supported TFC membranes fabricated using different heat-curing methods. The specific values of *J_w_* and *J_s_* were summarized in Table 5. Obviously, due to the probable defects in the non-curing PA layer, the water flux and reverse salt flux of the n-TFC FO membrane was not stable, which is relevant to its uneven surface morphology as observed in the SEM images and the incomplete polymerization verified by XPS and FTIR analysis. In comparison, the oven-curing and hot-water-curing TFC FO membranes exhibited much more stable water flux and reverse salt leakage. Specifically, the o-TFC membrane showed higher water fluxes and reverse salt fluxes in both FO and PRO modes, while w-TFC exhibited moderate water flux and significantly mitigated reverse salt diffusion (FO: 0.3 gMH; PRO: 0.8 gMH) because of the denser active layer with more crosslinked PA structure on the nanofiber support. *J_w_*/*J_s_*, which indicates the volume (L) of produced clean water along with certain reverse leakage (g) of draw solute, could provide a reasonable assessment of membrane separation efficiency in FO operation [47]. As shown in Figure 10c, the w-TFC FO membrane presented the best separation efficiency with the highest specific water flux (*J_w_*/*J_s_*) values (FO: 23.8 L/g; PRO: 12.9 L/g).

Therefore, when NaCl was used as a draw solute, hot-water-curing was proven to be truly preferable for the preparation of a nanofiber-supported TFC FO membrane with better selectivity. Furthermore, the FO performances of o-TFC and w-TFC over gradient concentration (0.5, 1, 1.5, 2 M) of NaCl draw solution were tested to acquire their intrinsic transfer properties, and the results are shown in Figure S3. It was found that w-TFC always exhibited acceptable water fluxes and superior salt rejections to o-TFC with increasing concentration of draw solution. It suggested that using a higher concentration of draw solution is expected to achieve a higher water flux while a still excellent salt rejection with the w-TFC membrane. The comparison of simulated water permeability (A, L m^−2^ h^−1^ bar^−1^) and salt permeability (B, L m^−2^ h^−1^) of o-TFC and w-TFC FO membranes are shown in Table S1. Notably, the o-TFC FO membrane showed nearly five times water permeability that of the w-TFC FO membrane, while the latter exhibited four times water/salt permeability that of the oven-curing TFC FO membrane.

High-rejection PA active layers would contribute to more stable water flux during FO operation since the osmotic pressure drop caused reverse salt leakage can be effectively reduced. As seen in Figure 10d, the water flux of the o-TFC FO membrane significantly declined due to its higher initial water flux and severer salt leakage, which would lead to the faster dilution of draw solution and ensuing lower osmotic pressure. In comparison, the w-TFC FO membrane with highly crosslinked PA layers and high rejection for NaCl draw solutes contributed to a more stable water flux for 16 h FO operation. The water flux decline ratio of the w-TFC FO membrane was only 10%, which was significantly superior to 40% of the o-TFC FO membrane. Therefore, hot-water-curing would be a preferable heat-curing process for constructing a durable FO membrane with stable water flux and excellent salt rejection.

Furthermore, considering the good water permeability and undesirable salt permeability (NaCl) of the o-TFC FO membrane, as well as the possible high rejection for divalent ions, MgCl_2_ was applied as a draw solute to get a better FO performance for the o-TFC FO membrane. It could be seen in Figure S4, both the o-TFC and w-TFC FO membranes operated under FO mode using 0.5 M MgCl_2_ as draw solution showed quite low reverse salt flux, while the o-TFC FO membrane performed with 2 times water flux of that of the w-TFC FO membrane. Therefore, oven-curing would be suggested to fabricate preferable nanofiber-supported TFC FO membranes for desalination of di- or multi-valent ions with proper draw solute, such as MgCl_2_.

### 3.6. Influence Mechanism of Heat Curing Method on the Formation of PA Active Layer

**Figure 11:**
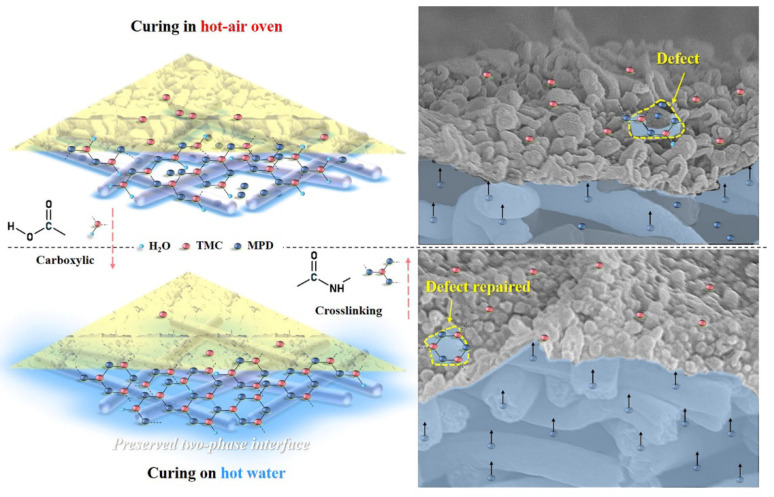
Schematic diagram of the formation process of nanofiber-supported PA-TFC FO membranes with oven-curing and hot-water-curing.

The influence mechanism of the heat-curing method on the formation processes of interfacially polymerized PA active layer on electrospun nanofiber support was deducted based on the experimental results mentioned above. Figure 11 illustrated the speculative formation process of PA active layers via interfacial polymerization of MPD in DI water with TMC in hexane using different heat-curing methods (curing in the hot-air oven and on hot water bath), which referred to the discussion in the literature [44]. PAN nanofiber exhibited good hydrophilicity with swelling behavior, which could first absorb an aqueous solution into the bulk or voids of the nanofiber support. Then, when organic TMC/hexane solution was poured onto the surface of nanofiber support soaked by MPD aqueous solution, the MPD molecules would diffuse into the organic phase and react with TMC molecules at the two-phase interface in a short time. After this, when the excess TMC/hexane solution was removed, the residual TMC could continue to react with the remaining MPD.

In the heat-curing step, on one hand, the interfacial polymerization could be accelerated and finalized by raising the reaction temperature, and on the other hand, the reaction could be terminated due to the evaporation of the rest solvents of hexane and water at the same time. Thus, heat-curing could be regarded as a necessary balance between the “repairing/healing” by further reaction of residual TMC with MPD and the termination of interfacial polymerization with the removal of excess hexane and water. Moreover, it is also effective to avoid the residual −COCl of TMC to react with water and produce linear carboxylic PA. After interfacial polymerization, the heat-curing above hot water could maintain an intact interface between the water with residual hexane, which is conducive for obtaining a highly crosslinked and less defective PA layer on the nanofiber support. More important, the aqueous monomer MPD in the nanofiber bulk could diffuse freely to have more chance to contact with the residual TMC. However, in the oven-curing process, water in the nanofiber support would evaporate quickly, which leads to the destruction of the water/organic two-phase interface, and the PA layer with defects tends to be formed, so the oven-curing TFC membrane showed higher reverse salt leakage.

Particularly, when the hyperbranched PEI with larger free volume was used as a substitute of MPD for fabricating a high-permeability NF-like PA layer, using oven-curing was also prone to get a more defective PA active layer. The preparation, characterization and FO performance of PEI/TMC-PA TFC membranes heat cured in an oven or on hot water were displayed and discussed in the Supporting Information (Figures S5–S7). It clearly showed that hot-water-curing could be expected to decrease membrane defects in the PA layer with certain aqueous monomers and improve the FO performance with appropriate draw solution.

## 4. Conclusions

In this work, it was proved that hot-water-curing (heat-curing on hot water with phase interface) could be considered as an effective way to “repair” or “heal” latent defects in the nanofiber-supported PA active layer. The further high-temperature interfacial polymerization during hot-water-curing enabled the PA active layer to have finer surface morphology, stronger superhydrophilicity, higher crosslinking degree and better tensile resistance. While in the oven-curing process, the quick collapse of the reaction interface due to the evaporation of solvent could contribute to the PA active layer with more linear carboxylic PA structures concaved into the nanofiber skeleton. Accordingly, in contrast with the oven-curing o-TFC FO membrane, the hot-water-curing endowed the w-TFC FO membrane with less reverse salt diffusion and higher selectivity under both FO and PRO modes when simulated seawater (3.5 wt %) was used as a draw solution. Notably, the o-TFC FO membrane showed advantages of higher water flux and comparable salt rejection compared to the w-TFC FO membrane when MgCl_2_ with divalent salt ions was used as a draw solution. Furthermore, the hot-water-curing nanofiber-supported TFC membrane with NF-like PA layer synthesized by hyperbranched PEI and TMC showed high water permeability and excellent selectivity with MgCl_2_ draw solution. In this preliminary study, the structure–activity relationship between physicochemical properties and separation performances of nanofiber-supported TFC FO membranes prepared with different heat-curing methods were revealed, and the formation mechanism of PA active layers on the electrospun nanofiber support was supposed. Deeper and further studies on the electrospinning support, monomers of interfacial polymerization, heat-curing processes, and draw solutes would greatly enhance fabricating high-performance electrospun nanofiber-supported TFC FO membranes for practical applications.

## Figures and Tables

**Table 1:** Surface roughnesses of the polyamide (PA) active layers of the TFC FO membranes prepared via different heat-curing methods.

Membrane	R_a_ (nm)	R_q_ (nm)	R_max_ (nm)
n-TFC	197	226	951
o-TFC	140	175	1128
w-TFC	168	211	1123

**Table 2:** Atomic compositions of nanofiber-supported PA layers prepared via different heat-curing methods.

Membrane	O%	N%	C%	O/N Ratio	Crosslinking Degree%
n-TFC	13.01	10.51	76.48	1.24	68.11
o-TFC	13.37	10.98	75.64	1.22	70.55
w-TFC	12.51	11.04	76.45	1.13	81.27

**Table 3:** Two curve-fitted peak components of high-resolution O 1s of nanofiber-supported PA layers prepared via different heat-curing methods.

Membrane	Carboxylic Oxygen (O=C‒O, 532.8 eV)%	Carbonyl Oxygen (O=C‒O/O=C‒N, 531.6 eV)%	Carboxylic/Carbonyl Ratio
n-TFC	42.38	57.62	0.74
o-TFC	39.83	60.17	0.66
w-TFC	31.78	68.22	0.47

**Table 4:** Two curve-fitted peak components of high-resolution O 1s of nanofiber-supported PA layers prepared via different heat-curing methods.

Membrane	1544 cm^−1^ (N−H)	1737 cm^−1^ (HO–C=O)	I_1544_/I_1__737_ Ratio
n-TFC	9.29	87.74	0.106
o-TFC	9.43	75.43	0.125
w-TFC	10.54	74.59	0.141

**Table 5:** Atomic compositions of nanofiber-supported PA layers prepared via different heat-curing methods.

Membrane	FO Mode			PRO Mode		
	*J_w_* (LMH)	*J_s_* (gMH)	*J_w_*/*J_s_* (L/g)	*J_w_* (LMH)	*J_s_* (gMH)	*J_w_*/*J_s_* (L/g)
n-TFC	8.0 ± 2.7	2.1 ± 1.0	4.6 ± 2.50	10.9 ± 0.45	3.6 ± 1.3	3.3 ± 1.09
o-TFC	12.3 ± 1.4	2.3 ± 0.3	5.5 ± 1.20	15.3 ± 0.5	2.2 ± 0.2	6.9 ± 0.33
w-TFC	6.7 ± 0.95	0.3 ± 0.03	23.8 ± 5.39	8.3 ± 0.6	0.8 ± 0.4	12.9 ± 7.68

## Data Availability

Not applicable.

## Supplementary Materials

The following are available online at https://www.mdpi.com/article/10.3390/membranes11040237/s1, Figure S1: Schematic diagram of the lab-scale FO setup. Figure S2: Water fluxes (*J_w_*) and reverse salt fluxes (*J_s_*) of the nanofiber supported TFC FO membranes prepared via hot-water-curing at different (a) curing temperatures; (b) curing time. Figure S3: Water fluxes (*J_w_*) and reverse salt fluxes (*J_s_*) of o-TFC and w-TFC with increasing NaCl concentration. Figure S4: Comparison of water fluxes (*J_w_*) and reverse salt fluxes (*J_s_*) of o-TFC and w-TFC under FO mode (Feed solution: DI; Draw solution: 0.5 M MgCl2). Figure S5: Comparison of water fluxes (*J_w_*) and reverse salt fluxes (*J_s_*) of the nanofiber supported PEI/TMC-PA TFC FO membranes prepared via hot-water-curing and oven-curing under FO mode (Feed solution: DI; Draw solution: 0.5 M MgCl_2_). Figure S6: SEM images of the PEI/TMC-PA layer and the nanofiber support of TFC FO membranes prepared via (a1, b1) oven-curing; (a2, b2) hot-water-curing: (a) active layer; (b) support layer. Figure S7: The changes of water contact angles (WCA) of nanofiber supported PEI/TMC-PA TFC FO membranes prepared via (a) oven-curing and (b) hot-water-curing over time. Table S1: Water Permeability and salt Permeability of o-TFC and w-TFC.
