# Supplementary material for: Alleviation of Reverse Salt Leakage across Nanofiber Supported Thin-Film Composite Forward Osmosis Membrane via Heat-Curing in Hot Water
Source: Membranes (Basel). 2021 Mar 27;11(4):237. doi: 10.3390/membranes11040237 (PMC8066147; doi:10.3390/membranes11040237)
Supplement: Supplementary file 1 [file membranes-11-00237-s001.pdf]

membranes

Article

Supplementary Materials: Alleviation of Reverse Salt Leakage Across Nanofiber Supported Thin-Film Composite Forward Osmosis Membrane via Heat-Curing in Hot Water

Xiao-Xue Ke [1,3], Ting-Yu Wang [1,3], Xiao-Qiong Wu [1,3], Jiang-Ping Chen [1,2,3], Quan-Bao Zhao [1,3] and Yu-Ming Zheng [1,2,3,*]

[1] CAS Key Laboratory of Urban Pollutant Conversion, Institute of Urban Environment, Chinese Academy of Sciences, 1799 Jimei Road, Xiamen 361021, China; xxke@iue.ac.cn (X.-X.K.); tywang@iue.ac.cn (T.-Y.W.); xqwu@iue.ac.cn (X.-Q.W.); jpchen@iue.ac.cn (J.-P.C.); qbzhao@iue.ac.cn (Q.-B.Z.)
[2] CAS Center for Excellence in Regional Atmospheric Environment, Institute of Urban Environment, Chinese Academy of Sciences, 1799 Jimei Road, Xiamen 361021, China
[3] University of Chinese Academy of Sciences, 19A Yuquan Road, Beijing 100049, China
* Correspondence: ymzheng@iue.ac.cn

Citation: Ke, X.-X.; Wang, T.-Y.; Wu, X.-Q.; Chen, J.-P.; Zhao, Q.-B.; Zheng, Y.-M. Alleviation of Reverse Salt Leakage Across Nanofiber Supported Thin Film Composite Forward Osmosis Membrane via Heat-Curing on Hot Water. *Membranes* **2021**, *11*, 237. https://doi.org/10.3390/membranes11040237

Academic Editor: Scott M. Husson

Received: 10 February 2021
Accepted: 24 March 2021
Published: 27 March 2021

Publisher's Note: MDPI stays neutral with regard to jurisdictional claims in published maps and institutional affiliations.

Copyright: © 2021 by the authors. Submitted for possible open access publication under the terms and conditions of the Creative Commons Attribution (CC BY) license (http://creativecommons.org/licenses/by/4.0/).

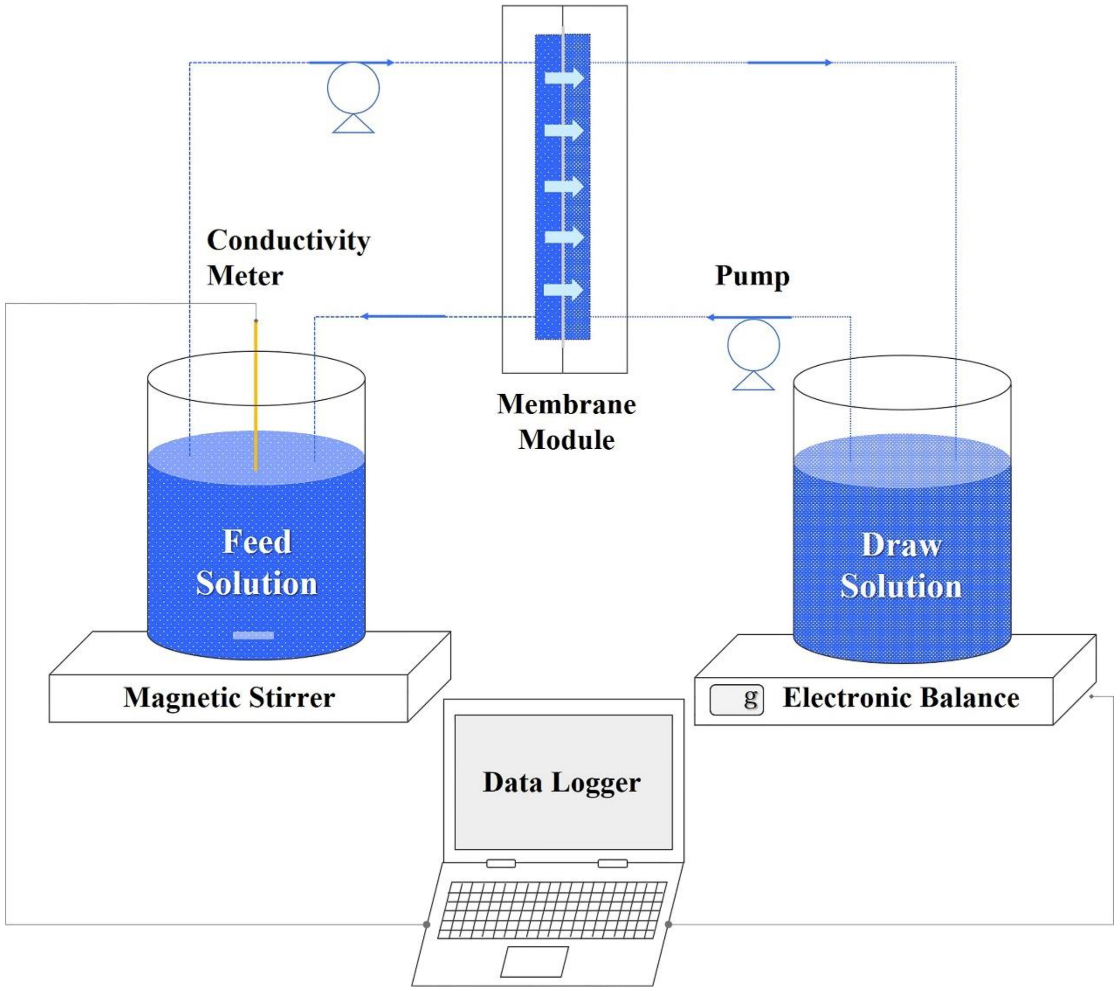

Figure S1. Schematic diagram of the lab-scale FO setup.

Optimization of the hot-water-curing condition

In order to explore the hot-water-curing condition for fabricating the PA active layer of nanofiber supported TFC membrane with optimum FO performance, the effects of curing temperatures (60, 70, 80, 90 ℃) and curing time (1, 2, 4, 6, 8, 10 min) on the pure water fluxes and reverse salt fluxes were investigated.

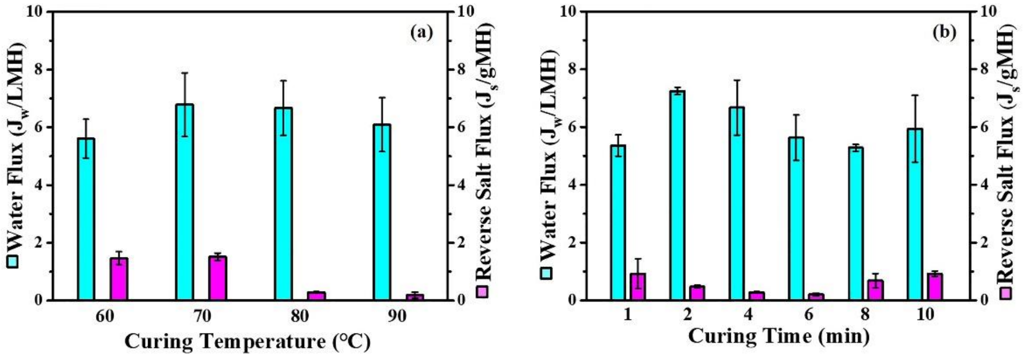

Figure S2. Water fluxes (J_w) and reverse salt fluxes (J_s) of the nanofiber supported TFC FO membranes prepared via hot-water-curing at different (**a**) curing temperatures; (**b**) curing time.

Theoretically, raising heat-curing temperature can accelerate the diffusion of monomers to the reaction zone, and promote the motion and crosslinking of PA chains. However, overlong heat treatment under high temperature would lead to the deformation, damage and delamination of the PA active layer on support. According the results in Figure S2a, the curing temperature exhibited an obvious impact on the salt rejection of w-TFC. It seemed that there are significant defects in PA cured on hot water below 80 ℃, as the as-prepared w-TFC showed much severer reverse salt leakage due to the incomplete polymerization with insufficient curing temperature. Furthermore, when curing at higher temperature (90 ℃), the obtained PA layer could be quite dense and thus showed decreased water flux. Additionally, as seen in Figure S2b, insufficient or excessive curing time could cause defects in hot-water-curing PA layer, and the proper curing time within 2-6 min range would be favorable for better FO performance. Based on the comprehensive consideration on the trade-off between water flux and reverse salt leakage, all the mentioned hot-water-curing w-TFC membranes in this paper were prepared conducting the curing parameter (80 ℃, 4 min) as default.

Intrinsic transfer parameters of o-TFC and w-TFC

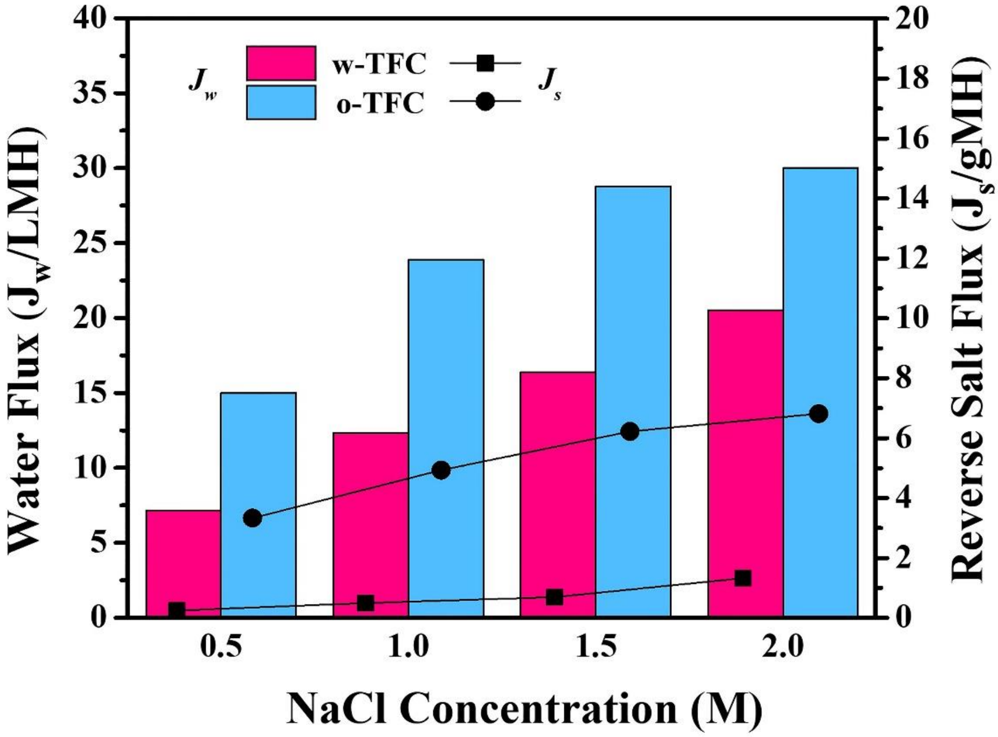

Figure S3. Water fluxes (J_w) and reverse salt fluxes (J_s) of o-TFC and w-TFC with increasing NaCl concentration.

Table S1. Water Permeability and salt Permeability of o-TFC and w-TFC.

Membrane	A, L $m^{-2}h^{-1}bar^{-1}$	B, L $m^{-2}h^{-1}$	A/B, bar^{-1}
**o-TFC**	0.85	0.19	4.5
**w-TFC**	0.18	0.01	18

Effect of the draw solute on FO performance of w-TFC

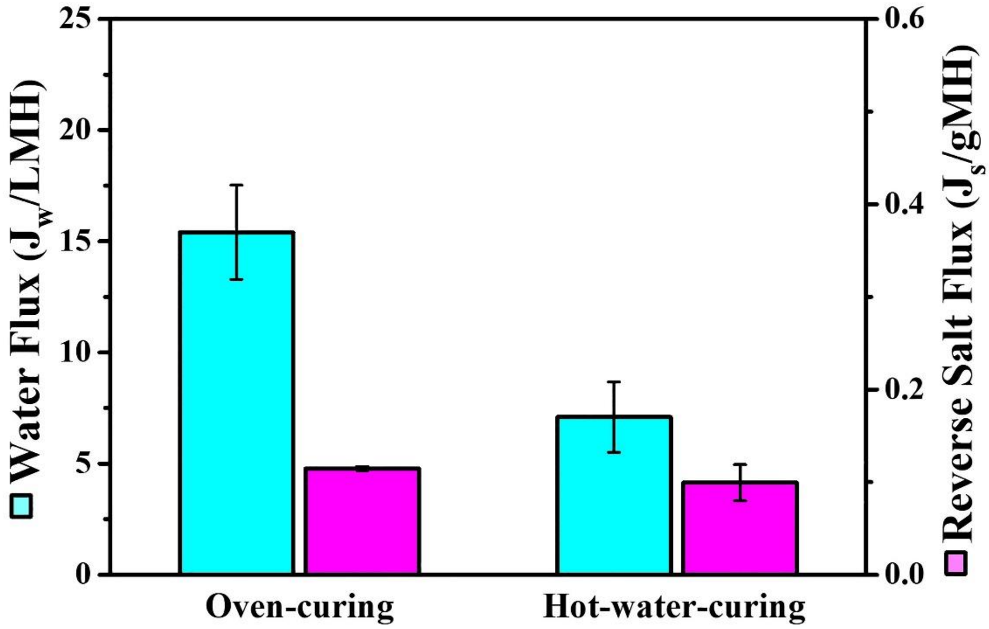

Figure S4. Comparison of water fluxes (J_w) and reverse salt fluxes (J_s) of o-TFC and w-TFC under FO mode (Feed solution: DI; Draw solution: 0.5 M $MgCl_2$).

Nanofiber supported PEI/TMC-PA TFC FO membrane

Typically, the MPD/TMC-PA film has inadequate hydrophilicity and rough ridge-valley morphology that is easily subjected to foulants blocking [1]. As it has been reported that, hyperbranched macromolecule polyethyleneimine (PEI) could be applied as a possible alternative to MPD, to fabricate loose and quite smooth hydrophilic nanofiltration (NF)-like PA active layers of TFC membranes with potential of high water permeability and low fouling propensity for FO application[2,3]. However, the oven-curing PEI/TMC-PA active layer possessing frequent defects, especially when prepared on the nanofiber support, hardly overcome the tradeoff between water flux and salt leakage. It normally showed severe salt leakage, or had to match with special draw solutes but showed relatively low water flux [4–7].

Herein, PEI was also employed to evaluate the "defect-healing" potential of hot-water-curing for the fabrication of the PEI/TMC-PA TFC membrane. The effects of adopting different heat curing methods on the morphology, hydrophilicity and FO performance of nanofiber supported PA layer synthesized by interfacial polymerization of PEI and TMC was further investigated.

Preparation process of the nanofiber supported PEI/TMC-PA TFC FO membrane

2% (w/v) PEI (Polyethyleneimine, Mw = 70000 g/mol, 50% aqueous solution, supplied by Aladdin, Shanghai, China) aqueous solution was used as an alternative monomer to MPD to saturate the PAN nanofiber support for 4 min, and then reacted with 0.2% (w/v)

TMC for 1 min to form a loose NF-like PA active layer on the nanofiber membrane, and then performed with heat curing in oven or on hot water at 80 ℃ for 4 min.

DI water and 0.5 M $MgCl_2$ were used as the feed solution and draw solution respectively to test the FO performance of the PEI/TMC-PA TFC membranes. And the other operation conditions were the same as that of MPD/TMC-PA TFC membranes.

FO performance of the nanofiber supported PEI/TMC-PA TFC FO membrane

As shown in Figure S5, the hot-water-curing PEI/TMC-PA TFC membranes exhibited comparable water flux, and quite lower reverse salt flux with excellent selectivity than the oven-curing TFC, which indicates the distinctive advantage of hot-water-curing over oven-curing in preparation of the PEI/TMC-PA active layer of TFC membrane with superior FO performance. Moreover, the formed PEI/TMC-PA possesses quite smooth morphology and superhydrophilicity (Figure S6, S7), which can be able to effectively avoid the blocking and accumulation of pollutants, compared to the ridge-valley microstructures of the MPD/TMC-PA layer. Therefore, with hot water as the heat curing medium, PEI would be a good alternative as the aqueous monomer for interfacial polymerization thus achieving the aim of fabricating high-performance TFC FO membranes based on nanofiber support possessing favorable surface morphology and property for antifouling and cleaning. Simultaneously, with proper selection of novel draw solutes [8–10], heat curing would be a facile way to tune the surface properties and FO performance of nanofiber supported PA-TFC membranes for specific applications.

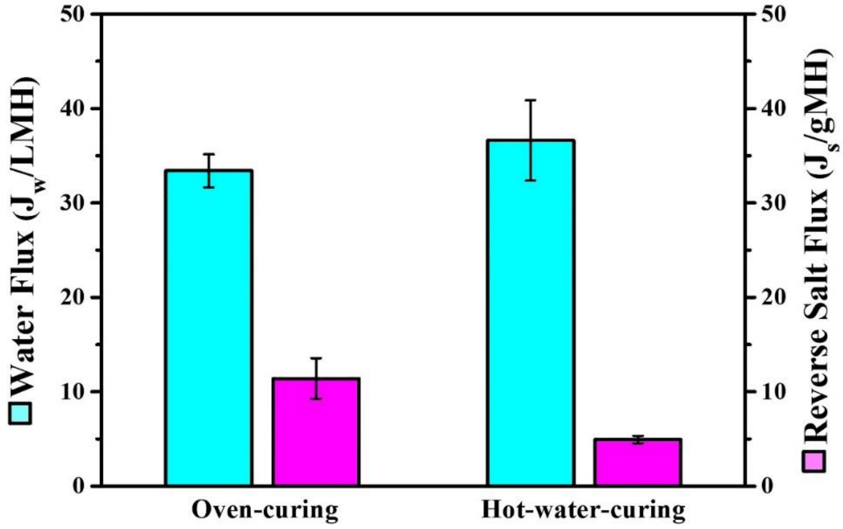

Figure S5. Comparison of water fluxes (J_w) and reverse salt fluxes (J_s) of the nanofiber supported PEI/TMC-PA TFC FO membranes prepared via hot-water-curing and oven-curing under FO mode (Feed solution: DI; Draw solution: 0.5 M $MgCl_2$).

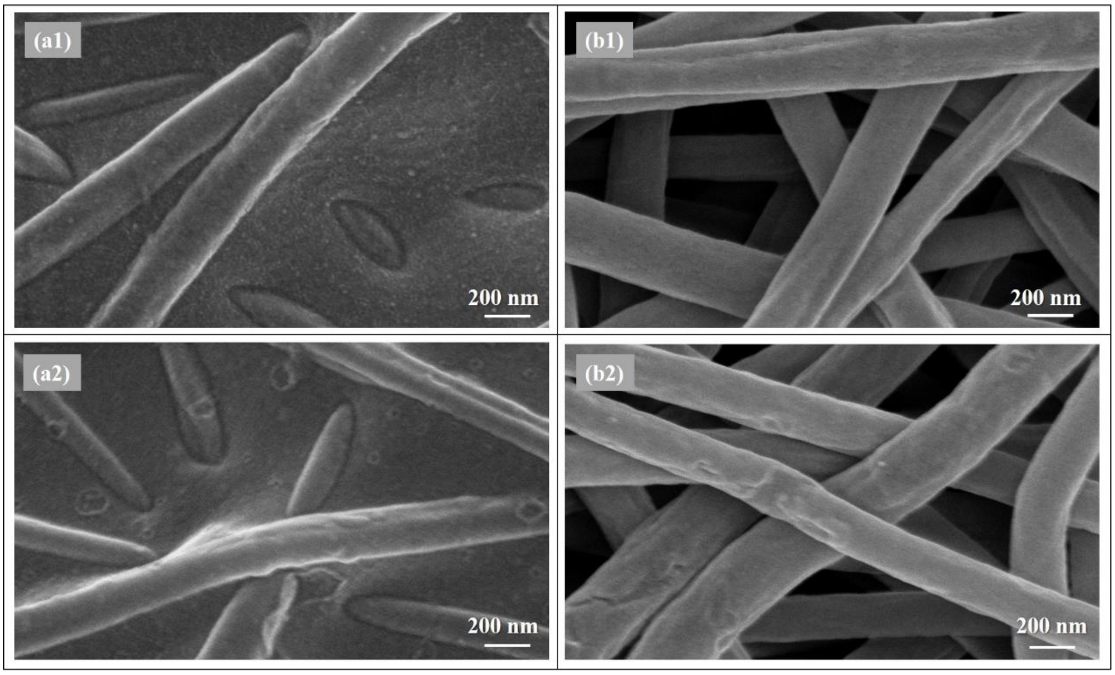

Figure S6. SEM images of the PEI/TMC-PA layer and the nanofiber support of TFC FO membranes prepared via (**a**1, **b**1) oven-curing; (**a**2, **b**2) hot-water-curing: (**a**) active layer; (**b**) support layer.

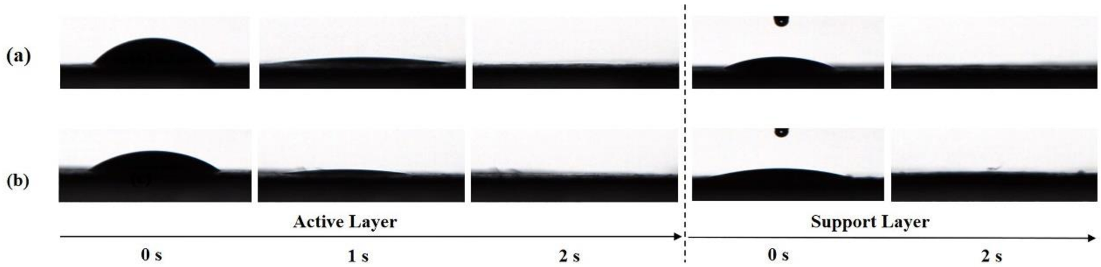

Figure S7. The changes of water contact angles (WCA) of nanofiber supported PEI/TMC-PA TFC FO membranes prepared via (**a**) oven-curing and (**b**) hot-water-curing over time.

References

1. Shang, C.; Pranantyo, D.; Zhang, S. Understanding the Roughness–Fouling Relationship in Reverse Osmosis: Mechanism and Implications. Environ. Sci. Technol. **2020**, *54*, 5288–5296. https://doi.org/10.1021/acs.est.0c00535.
2. Gohil, J.M.; Ray, P. A review on semi-aromatic polyamide TFC membranes prepared by interfacial polymerization: Potential for water treatment and desalination. Sep. Purif. Technol. **2017**, *181*, 159–182. https://doi.org/10.1016/j.seppur.2017.03.020.
3. Wei, X.; Hong, J.; Zhu, S.; Chen, J.; Lv, B. Structure–performance study of polyamide composite nanofiltration membranes prepared with polyethyleneimine. J. Mater. Sci. **2017**, *52*, 11701–11714. https://doi.org/10.1007/s10853-017-1225-0.
4. Bui, N.N.; McCutcheon, J.R. Nanofiber supported thin-film composite membrane for pressure-retarded osmosis. Environ. Sci. Technol. **2014**, *48*, 4129–4136. https://doi.org/10.1021/es4037012.
5. Cho, M.; Lee, S.H.; Lee, D.; Chen, D.P.; Kim, I.-C.; Diallo, M.S. Osmotically driven membrane processes: Exploring the potential of branched polyethyleneimine as draw solute using porous FO membranes with NF separation layers. J. Membr. Sci. **2016**, *511*, 278–288. https://doi.org/10.1016/j.memsci.2016.02.041.
6. Xu, S.; Li, F.; Su, B.; Hu, M.Z.; Gao, X.; Gao, C. Novel graphene quantum dots (GQDs)-incorporated thin film composite (TFC) membranes for forward osmosis (FO) desalination. Desalination. **2019**, *451*, 219–230. https://doi.org/10.1016/j.desal.2018.04.004.
7. Hamid, M.F.; Abdullah, N.; Yusof, N.; Ismail, N.M.; Ismail, A.F.; Salleh, W.N.W.; Jaafar, J.; Aziz, F.; Lau, W.J. Effects of surface charge of thin-film composite membrane on copper (II) ion removal by using nanofiltration and forward osmosis process. J Water Process Eng. **2020**, *33*, 101032. https://doi.org/10.1016/j.jwpe.2019.101032.
8. Ge, Q.; Ling, M.; Chung, T.-S. Draw solutions for forward osmosis processes: Developments, challenges, and prospects for the future. J. Membr. Sci. **2013**, *442*, 225–237. https://doi.org/10.1016/j.memsci.2013.03.046.
9. Liao, X.; Zhang, W.-H.; Ge, Q. A cage-like supramolecular draw solute that promotes forward osmosis for wastewater remediation and source recovery. J. Membr. Sci. **2020**, *600*, 117862. https://doi.org/10.1016/j.memsci.2020.117862.

10. Khraisheh, M.; Gulied, M.; AlMomani, F. Effect of Membrane Fouling on Fertilizer-Drawn Forward Osmosis Desalination Performance. Membranes. **2020**, *10*, 243.
